# The relationship between dietary phytochemical index and resting metabolic rate mediated by inflammatory factors in overweight and obese women: a cross-sectional study

**DOI:** 10.1186/s12905-022-01894-9

**Published:** 2022-07-25

**Authors:** Atieh Mirzababaei, Akram Taheri, Niloufar Rasaei, Sanaz Mehranfar, Shahin Jamili, Cain C. T. Clark, Khadijeh Mirzaei

**Affiliations:** 1 Department of Community Nutrition, School of Nutritional Sciences and Dietetics, Tehran University of Medical Sciences (TUMS), Tehran, Iran; 2 Department of Nutrition, Faculty of Medicine, Sciences and Research Branch, Islamic Azad, University, Tehran, Iran; 3 Department of Surgery, Shahid Beheshti University of Medical Sciences, Tehran, Iran; 4 Centre for Intelligent Healthcare, Coventry University, Coventry, U.K.; 5 Food Microbiology Research Center, Tehran University of Medical Sciences, Tehran, Iran

**Keywords:** Resting metabolic rate, Obesity and overweight, Dietary phytochemical index, Inflammatory marker

## Abstract

**Background:**

Unhealthy dietary patterns are the most important modifiable risk factors for obesity and overweight**.** This study aimed to examine the relationship between Dietary Phytochemical Index (DPI) and resting metabolic rate (RMR), mediated by inflammatory factors, in overweight and obese women.

**Methods:**

A total of 404 women, aged 18–48 years, were included in the cross-sectional study. DPI was calculated using the 147-item food frequency questionnaire (FFQ). Anthropometric measurements, RMR, and blood biomarkers were assessed using standard protocols.

**Results:**

There was marginally significant association between adherence to DPI and RMR status in the crude model (OR = 1.41, 95% CI 0.94–2.11, *P* = 0.09). After adjusting for potential confounders, a significant association was seen between the DPI and increase RMR.per.kg (OR = 2.77, 95% CI 0.98–7.82, *P* = 0.05). Our results indicated that plasminogen activator inhibitor-1 (PAI-1), transforming growth factor (TGF-β), and monocyte chemoattractant protein-1 (MCP-1) had a mediatory effect on the association between RMR and DPI (*P* > 0.05). Indeed, it was shown that, PAI-1, TGF-β, and MCP-1 destroyed the significance of this association and could be considered as mediating markers. However, no mediating effect was observed for high-sensitivity C reactive protein (hs-CRP).

**Conclusions:**

Adherence to DPI can improve the RMR by reducing levels of inflammatory markers, and may be considered as a treatment for obesity. However, more long-term studies are recommended.

## Introduction

Incidence of overweight and obesity (defined as 25 $\le \hspace{0.17em}$BMI $\le \hspace{0.17em}$29.9 kg/m^2^, and BMI $\ge \hspace{0.17em}$30 kg/m^2^ respectively) represents an ongoing epidemic, are important metabolic diseases, and the main reason for the increase in mortality worldwide. Obesity is defined as an excessive increase in adipose tissue, that may increase the risk of other chronic diseases, such as diabetes mellitus, cardiovascular disease (CVD), inflammatory disorders, high blood pressure, cancers, etc. [1]. According to the World Health Organization (WHO), the prevalence of obesity is 21% and overweight is 53%, and it is predicted that by the year 2030, the population of obese and overweight people will increase [2, 3]. The prevalence of obesity and overweight is common in women, possibly due to more adipose tissue, decreased physical activity, decreased resting metabolic rate (RMR), and improper eating pattern, etc. [4]. Excessive triglyceride (TG) accumulation in adipocytes contributes to adipocyte hypertrophy and increases the expression of chemokines such as monocyte chemoattractant protein-1 (MCP-1) and inflammatory cytokines such as plasminogen activator inhibitor type-1 (PAI-1), transforming growth factor-beta (TGF-β), high-sensitivity C-reactive protein (hs-CRP), and infiltration of immune cells into fat cells and the development of inflammatory responses, specifically around intra- abdominal adipose tissue, which leads to the decrease of RMR [5]. Indeed, empirical studies show that a lower RMR is commonly seen in people with obesity [6, 7]. Several factors, including gender, age, weight, fat-free mass (FFM), body fat, and body fat distribution can affect RMR [8, 9]. Factors such as deregulation in energy, behavioral factors, changes in diet, age, sex, changes in activity level and environmental factors can contribute to increasing obesity [10]. Moreover, studies show that the number of phytochemicals in all plant foods can reduce the underlying disorders of CVD, diabetes, cancer, obesity, and overweight [11–15]. Phytochemicals include phenolic compounds (flavonoids, phenolic acids, hydroxycinnamic acids, lignans, stilbenoids, isoprenoids), organosulphur compounds (allyl sulfurs, isothiocyanates) and carotenoids (beta-carotene, lycopene, lutein, zeaxanthin) are natural bioactive compounds found abundantly in fruits, vegetables, whole grains, legumes, seeds, nuts, spices, soybeans and soybeans products, cocoa and their derived beverages such as coffee, tea and wine [16, 17]. Polyphenols in fruits and vegetables, soybeans and soybean products, spices, coffee and tea, such as quercetin, naringenin, routine, hesperidin, resveratrol, anthocyanin, isoflavones, phytosterol, cinnamon, caffeine, and catechins, can reduce appetite, regulate the metabolism of carbohydrates and lipids, and increase lipid peroxidation and fatty acid mitochondrial beta-oxidation, all of which play an important role in reducing the accumulated fat in the body [18, 19]. Antioxidants, dietary phytochemicals, polyphenols, and other components in the DPI stimulate the sympathetic system, therefore, increasing thermogenic consequently affects RMR [20–22]. The aforementioned factors also reduce inflammatory factors by reducing reactive oxygen (ROS) and inhibiting cyclooxygenase 2 [19, 23]. Vincent et al. showed that DPI was inversely related to body fat mass and oxidative stress [23]; in addition, DPI based on the percentage of daily energy intake of foods rich in phytochemicals is related to the total energy intake[24, 25]. According to this index, vegetarian diets will have a score of 100 and western diets will have a score of less than 20 [26]. This index is a simple method to assess the intake of phytochemicals in the daily diet and despite some limitations, it can have clinical applications [27]. The study of the relationship between DPI and RMR and the mediating effect of inflammatory factors among obese and overweight women is important, particularly because RMR is low in obese and overweight women, which may be due to the mediating effect of inflammatory factors [28, 29]. For this reason, this study evaluated the association between DPI and RMR, mediated by inflammatory factors (MCP-1, PAI-1, TGF-β and hs-CRP), in overweight and obese women.

## Materials and methods

### Study population

In this cross-sectional study, 404 overweight and obese women were recruited between 2017 to 2019, from health centers of all the regions of West and Central Tehran, using community-based comfort sampling. The protocol was accepted by Ethical Commission at Tehran University of Medical Science (IR.TUMS.VCR.REC.1395.1597). All methods were performed in accordance with the declaration of Helsinki. This study was supported by grants from the TUMS (Grant’s ID: 97-03-161-41,155). All participants were invited to the nutritional laboratory of Tehran University of Medical Sciences and all signed a written informed consent prior to participation in the study. The study participants were selected based on pre-defined inclusion criteria, body mass index (BMI 25–40 kg/m^2^), aged 18–48 years, an absence of any acute or chronic inflammatory disease, no history of hypertension, no alcohol or drug abuse, and not being pregnant. Accordingly, participants who had a history of diabetes mellitus, stroke, kidney disease, and cancer, cardiovascular and thyroid diseases were excluded from the study. Furthermore, we excluded those women with weight loss history in recent years, menopause, smokers, and those who reported total daily energy lower than 800 kcal/d (3347 kJ/d) or higher than 4200 kcal/d (17,573 kJ/d) [30].

### Anthropometric measurements

Body weight was measured with a calibrated digital scale to the nearest 0.1 kg with minimum of clothes and without shoes. Standing height was measured with an accuracy of 0.5 cm without shoes, using a tape measure. BMI was calculated by weight (kg)/ height (m) squared. A non-elastic tape was used to assess waist and hip circumferences to the nearest 1.0 cm. These measurements were performed in compliance with WHO recommendations [31]. All measurements were performed by one person to reduce the measurement errors. According to the definition of the world health organization, overweight as 25 ≤ BMI ≤ 29.9 kg/m^2^ and obesity is grading Grade 1, 2 and 3 as 30 ≤ BMI ≤ 34.9 kg/m^2^, 35 ≤ BMI ≤ 39.9 kg/m^2^ and BMI ≥ 40 kg/m^2.^

### Biochemical evaluation

Fasting blood samples were collected after 12–14 h from all study participants. The serum was centrifuged and stored at a temperature of − 80 C. Fasting serum glucose, triglyceride levels, total cholesterol levels and direct high density lipoprotein-cholesterol were measured by the GOD/PAP method, GPO–PAP method, Enzymatic Endpoint method and enzymatic clearance assay, respectively. Serum hs-CRP was measured by the use of an immunoturbidimetric assay (high-sensitivity assay, Hitachi 902) and for other inflammatory factors (MCP-1, PAI-1 and TGF-β) the ELISA kit was used (AccuBind, Monobind Inc., USA).

### Dietary assessment and phytochemical index calculation

Dietary intake was assessed by a validated and reliable semi-quantitative 147-item food frequency questionnaire (FFQ) that has been validated in previous work [32]. Items are defined based on a series of foods or beverages which are categorized into 9 major food groups. Food frequency categories, “daily/weekly/monthly”, were used, and participants were asked to report their consumption frequency of each food item. FFQ data were analyzed using nutritionist 4software, and used to compute energy and nutrient content of foods based on united states department of agriculture (USDA) food composition table, modified for Iranian foods [33, 34]. Dietary data were analyzed by an expert professional using FFQ. The DPI was proposed and calculated by McCarty. {DPI = [daily energy derived from phytochemical- rich foods kJ (kcal)/ total daily energy intake kJ (kcal)] × 100} [26, 35, 36].

### IPAQ assessment

Physical activity (PA) levels were measured using the international physical activity questionnaire-short form (IPAQ), which also included normal activities of daily living, frequency, and time spent each week for that activity during the last 12 months. The level of physical activity was expressed as metabolic equivalent hours per week (METs-h/week) [37].

### Resting metabolic rate measurement

RMR was measured by indirect calorimetry using a metabolic cart (MetaLyzer^®^3B, made in Germany). The RMR was assessed in the morning after a requested overnight fast (10–12 h). The indirect calorimetry device was calibrated before each assessment. Before the test, participants had to rest in a supine position for 10–20 min to create relatively stable conditions. Further, the first 5 min were not considered for analysis to ensure a stable condition, and only the final 15 min were used to calculate the RMR [38]. Patients were required to avoid nicotine, caffeine, and exercise for at least 4 h before the calorimetry assessment [33, 39]. Based on a previous study, we considered the cutoff for dichotomization to be 20 kcal/24 h/kg, which has been posited to predict the risk of obesity and its complications [40]. Accordingly, participants were classified into two groups, group 1 (n = 171) who had low RMR/kg (< 20 kcal/24 h/kg), and group 2 (n = 233), who had high (RMR/kg > 20 kcal/24 h/kg).

### Statistical analyses

The Kolmogorov- Smirnov test was used to evaluate the normality of the data. All statistical analysis was conducted using SPSS version 23.0 (SPSS, Chicago, IL, 131 US). Quantitative variables were presented as mean and SD and qualitative variables were reported in percentages. The relationship between quantitative variables among high and low RMR.per.kg and DPI was assessed using independent-sample t-tests and re-analyzed using analysis of covariance (ANCOVA) to adjust the effects of confounders, including age, BMI, energy intake, and physical activity. Binary logistic regression was used to discern the mediating role of inflammatory factors on the relationship between RMR.per.kg and DPI. Low adherence to DPI and RMR.per.Kg < 20 kcal/24 h/kg represented our reference group. In model 1, the effect of age, waist to hip ratio (WHR), energy intake, and physical activity were adjusted, and in model 2, all components of food which were significant after adjustment for energy intake, marital status, housing status, job, weight loss history in past years, economic status, number of family members, housing situation, blood pressure, and education, were also added to model 1. To estimate the mediatory role of inflammatory factors, we added the final modified model to the complete model. If inflammatory factors attenuated significance, they were considered as mediators, but if a significant relationship remained, they were regarded as having no mediating effect. In the present study P < 0.05 was a priori considered statistically significant.

## Results

### Study population characteristics

The mean age, weight, BMI, RMR.per.kg, RMR, WC, and WHR of 404 women who were participating in our study were 36.72 (SD = 9.10) years, 81.29 (SD = 12.43) kg, 31.26 (SD = 4.29) kg/m2, 19.59 (SD = 3.09) kcal/24 h/kg, 1574.96 (SD = 259.71) kcal/day, 99.61 (SD = 10.07) cm, and 1.16 (SD = 4.54) cm, respectively. The percentage of participants that were illiterate, had a diploma, and > Diploma was 0.9%, 12.12%, and 86.88%, respectively.

### Characteristics of study population based on RMR.per.kg categorization

To examine the association of biochemical variables, body composition and serum inflammatory marker levels with RMR.per.kg, the individuals were grouped based on RMR /kg; group 1 with low RMR/kg (< 20 kcal/24 h/kg) and group 2 with high RMR/kg (≥ 20 kcal/24 h/kg) (n = 171 and n = 233 respectively), (Table 1). The results showed that there were significant differences in BMI (*P* < 0.0001), weight (*P* < 0.0001), LDL (*P* = 0.05), WC (*P* < 0.0001), RMR (*P* < 0.0001) and marital status (*P* = 0.01) between people with RMR/kg < 20 kcal/24 h/kg, and RMR/kg ≥ 20 kcal/24 h/kg. After adjusting for age, BMI, PA (physical activity) and energy intake a significant difference was seen between weight (*P* < 0.0001), WC (*P* < 0.0001), WHR (*P* < 0.0001), TG (*P* = 0.04), RMR (*P* = 0.001), DBP (*P* = 0.009), SBP (*P* = 0.03) and job (*P* = 0.02) between two groups.

**Table 1:** Characteristics of study population based on RMR categorization

Variables	RMR.per.kg < 20 kcal/24 h/kg (n = 171) Mean ± SD	RMR.per.k ≥ 20 kcal/24 h/kg (n = 233) Mean ± SD	0.95% CI	*P*-value	*P*-value*
*Quantitative variables*					
Demography					
Age (years)	37.50 ± 8.02	36.06 ± 9.78	− 0.36, 3.25	0.11	0.16
Body composition					
BMI (kg/m^2)^	32.3 ± 4.49	30.47 ± 3.97	1.01, 2.69	** < 0.0001**	0.73
Weight (kg)	83.96 ± 13.53	79.30 ± 11.16	2.22, 7.08	** < 0.0001**	** < 0.0001**
Height (cm)	160.98 ± 5.96	161.40 ± 5.80	−1.58, 0.75	0.48	0.07
WC (cm)	102 ± 10.36	97.86 ± 9.50	2.17, 6.10	** < 0.0001**	** < 0.0001**
WHR	1.48 ± 7.00	0.92 ± 0.05	−0.34, 1.45	0.22	** < 0.0001**
Biochemical measurements					
FBS (mmol/L)	88.0 ± 10.12	86.71 ± 8.90	1.09, 3.73	0.28	0.42
TG (mmol/L)	125.77 ± 63.37	113.72 ± 55.88	−3.06, 27.16	0.13	**0.04**
T-chol (mmol/L)	186.71 ± 35.46	183.26 ± 36.27	−5.51, 12.4	0.45	0.22
LDL (mg/dL)	97.68 ± 24.85	91.86 ± 22.71	−0.18, 11.82	**0.05**	0.62
HDL-C (mg/dL)	45.64 ± 10.96	47.93 ± 10.62	−5.00, 0.41	0.09	0.49
Blood pressure					
SBP (mmHg)	111.94 ± 13.91	110.66 ± 15.91	− 2.18, 4.73	0.47	**0.03**
DBP (mmHg)	77.37 ± 9.07	77.91 ± 11.93	− 2.97, 1.89	0.67	**0.009**
Inflammatory factors					
PAI-1 (mg/dI)	18.04 ± 32.11	13.73 ± 27.03	− 4.82, 13.45	0.34	0.65
MCP-1 (mg/dI)	54.33 ± 102.47	41.89 ± 71.28	− 11.57, 36.4	0.27	0.42
hs-CRP (mg/L)	4.77 ± 4.62	3.73 ± 4.57	− 0.13, 2.22	0.08	0.65
TGF-β (mg/L)	83.24 ± 59.31	73.29 ± 29.87	− 5.03, 24.94	0.19	0.46
RMR measurements					
RMR (Kcal)	1482.52 ± 254.03	1701.42 ± 209.97	− 273.66, 164.5	** < 0.0001**	**0.001**
RMR.per.kg	17.56 ± 1.88	22.37 ± 2.11	− 5.27, − 4.35	** < 0.0001**	** < 0.0001**
Physical activity					
Physical activity	1145.82 ± 1759.29	1310.58 ± 2519.97	823.52,1479.08	0.54	0.60
*Qualitative variables*					
Education					
Illiterate	2 (50.0)	2 (50.0)	**–**	0.22	0.69
Diploma	26 (53.1)	23 (46.9)			
> Diploma	143 (40.75)	208 (59.25)			
*Marital status*					
Married	131 (45.8)	155 (54.2)	–	**0.01**	0.81
Single	40 (33.9)	78 (66.1)			
Job					
Unemployed	111 (44.4)	139 (55.6)	–	0.27	**0.02**
Employed	60 (38.96)	94 (61.04)			
Housing status					
Landlord	97 (40.9)	140 (59.1)	–	0.61	0.91
Leased	74 (44.32)	93 (55.68)			
Number of family members					
< 4	137 (42.0)	189 (58.0)	**–**	0.28	0.61
** ≥ 4**	34 (43.59)	44 (56.41)			
Weight loss history in past years					
Yes	82 (41.8)	114 (58.2)	–	0.26	0.60
No	89 (42.79)	119 (57.21)			
Economic status					
Low	16 (40.0)	24 (60.0)	–	0.64	0.76
Moderate	75 (44.91)	92 (55.09)			
High	60 (38.7)	95 (61.3)			
Very high	20 (47.62)	22 (52.38)			

Data are presented as Mean ± SD or number (percent)

Bold: *P* value ≤ 0.05

*BMI* Body mass index, *WHR* Waist, hip ratio, *WC* Waist circumference, *FBS* Fasting blood sugar, *TG* Triglyceride, *T-chol* Total cholesterol, *LDL* Low-density lipoprotein, *HDL-C* High density lipoprotein cholesterol, *DBP* Diastolic blood pressure, *SBP* Systolic blood pressure, *PAI-1* Plasminogen activator inhibitor type-1, *MCP-1* Monocyte chemoattractant protein-1, *hs-CRP* High-sensitivity C-reactive protein, *TGF-β* Transforming growth factor beta, *RMR* Resting metabolic rate

*P*-value was for t test and *P*-value* was for ANCOVA, adjusted for age, BMI, physical activity, energy, *P ≤ 0/05 is significant

### Dietary intake of study population based on RMR.per.kg categorization

The dietary intake of the participants across two groups of RMR.per.kg is shown in Table 2. After adjustment for energy intake, seen significant differences in dietary fibers intake (*P* = 0.005), glucose (*P* = 0.01), fructose (*P* = 0.005), sucrose (*P* = 0.007), and olive (*P* = 0.02) between the two groups.

**Table 2:** Dietary intake of study population based on RMR.per.kg categorization

Variables	RMR.per.kg < 20 kcal/24 h/kg (n = 171) Mean ± SD	RMR.per.kg ≥ 20 kcal/24 h/kg (n = 233) Mean ± SD	*P*-value	*P*-value*
Energy (kcal/d)	2566.26 ± 767.68	2680.19 ± 835.86	0.17	–
Carbohydrate (g/d)	365.68 ± 122.51	377.18 ± 126.08	0.36	0.30
Protein (g/d)	87.50 ± 30.12	93.97 ± 32.16	**0.04**	0.12
Fat (g/d)	**93.54 ± 36.86**	**105.01 ± 32.44**	**0.52**	**0.32**
Total fiber (g/d)	43.44 ± 18.01	50.07 ± 23.06	**0.002**	**0.005**
*Carbohydrates subgroups*				
Glucose (g/d)	20.67 ± 12.69	19.39 ± 9.05	0.26	**0.01**
Fructose (g/d)	25.02 ± 14.78	23.06 ± 10.77	0.13	**0.005**
Sucrose (g/d)	33.42 ± 23.45	30.03 ± 16.90	0.09	**0.007**
*Fatty acids*				
Polyunsaturated fatty acids (g/d)	19.61 ± 9.70	20.40 ± 9.47	0.42	0.96
Monounsaturated fatty acids (g/d)	30.72 ± 12.31	32.90 ± 13.27	0.10	0.35
Linoleic (g/d)	16.86 ± 9.15	17.77 ± 8.87	0.33	0.81
Linolenic (g/d)	1.21 ± 0.71	1.21 ± 0.063	0.96	0.37
*DPI Components*				
Vegetables(g/d)	442.47 ± 281.84	413.63 ± 249.03	0.29	0.28
Fruits(g/d)	521.56 ± 333.42	484.64 ± 334.33	0.28	0.17
Legumes(g/d)	44.20 ± 34.17	41.77 ± 38.43	0.51	0.83
Whole grains(g/d)	69.98 ± 67.31	67.77 ± 80.78	0.76	0.73
Nuts and Seeds(g/d)	15.66 ± 16.18	14.89 ± 20.58	0.68	0.88
Olive (g/d)	5.65 ± 8.49	4.24 ± 5.58	**0.04**	**0.02**
Soy(g/d)	4.6 ± 7.72	4.84 ± 7.73	0.84	0.89
Coffee	0.48 ± 1.10	0.53 ± 1.00	0.65	0.62
Tea	7.48 ± 5.78	6.96 ± 8.38	0.46	0.25
Spices(g/d)	3.28 ± 2.08	3.36 ± 2.17	0.68	0.83

Bold: *P* value ≤ 0.05

^*^After adjustment for calorie intake

DPI, deitary phychemical index

*P ≤ 0/05 is significant

### Characteristics of the study population according to categorized DPI

Participants were divided into two groups based on median intake DPI. General characteristics of participants, such as body composition, blood pressure, biochemical assessment, Inflammatory factors, RMR measurement, qualitative variable and others among lower vs. higher than the median of intake DPI, are presented in Table 3. After adjusting for potential confounders the results showed that there was a significant mean difference among DPI categories for RMR.per.kg (*P* < 0.0001) and there was a significant difference between two groups for economic status (*P* < 0.05) (Table 3).

**Table 3:** Characteristics of study population between high and low DPI adherence

Variables	Low adherence (n = 171) Mean ± SD	high adherence (n = 233) Mean ± SD	*P*-value	*P*-value*
*Quantitative variables*				
Age (years)	36.25 ± 9.41	37.17 ± 8.80	0.31	0.12
Body composition				
BMI (kg/m^2)^	31.41 ± 4.44	30.90 ± 3.99	0.23	0.30
Weight (kg)	81.92 ± 13.21	80.19 ± 11.23	0.16	0.18
Height (cm)	161.23 ± 5.78	161.28 ± 5.99	0.93	0.97
WC (cm)	99.96 ± 10.47	9.5098.94 ± 9.50	0.31	0.21
WHR	0.93 ± 0.05	1.40 ± 6.52	0.32	0.21
Biochemical measurements				
FBS (mmol/L)	9.22 ± 87.60	87.14 ± 10.25	0.71	0.47
TG (mmol/L)	115.33 ± 56.88	119.40 ± 59.31	0.58	0.71
T-chol (mmol/L)	184.88 ± 34.12	185.76 ± 36.92	0.84	0.73
HDL-C (mg/dL)	45.97 ± 10.93	47.64 ± 10.69	0.22	0.22
LDL (mg/dL)	94.75 ± 22.11	96.31 ± 26.19	0.60	0.49
Blood pressure				
SBP (mmHg)	110.85 ± 13.17	111.89 ± 16.24	0.55	0.41
DBP (mmHg)	77.26 ± 9.76	77.89 ± 11.19	0.61	0.27
Inflammatory factors				
PAI-1 (mg/dI)	17.73 ± 33.13	14.39 ± 26.37	0.47	0.48
MCP-1 (mg/dI)	54.33 ± 102.47	41.89 ± 71.28	0.15	0.31
hs-CRP (mg/L)	4.73 ± 5.09	3.74 ± 3.78	0.98	0.07
TGF-β (mg/L)	83.43 ± 58.80	73.43 ± 34.98	0.18	0.15
RMR measurements				
RMR	280.04 ± 1592.3	1556 ± 237.42	0.23	0.95
RMR.per.kg	19.58 ± 3.05	3.18 ± 19.69	0.77	** < 0.0001**
*Qualitative variable*				
Education				
Illiterate	3 (75.0)	1(25.0)	0.31	0.69
Diploma	20 (41.67)	28 (58.33)		
> Diploma	148(42.05)	204(57.95)		
Marital status				
Married	53 (51.5)	50 (48.5)	0.64	0.69
Single	118 (39.219)	183 (60.79)		
Job				
Unemployed	120 (49.6)	122 (50.4)	0.93	0.98
Employed	51 (31.49)	111 (68.51)		
Housing status				
Landlord	124 (54.4)	104 (45.6)	**0.04**	0.28
Leased	47 (26.71)	129 (73.29)		
Number of family members				
< 4	155 (48.9)	162 (51.1)	0.28	**0.05**
** ≥ 4**	16 (18.4)	71 (81.6)		
Weight loss history in past years				
Yes	112 (58.6)	79 (41.4)	**0.003**	0.88
No	59 (27.7)	154 (72.3)		
Economic status				
Low	17 (44.7)	21 (55.3)	0.25	**0.01**
Moderate	78 (48.1)	84 (51.9)		
High	70 (40.7)	102 (59.3)		
Very high	6 (18.75)	26 (81.25)		

Data are presented as Mean ± SD or number (percent)

Bold: *P* value ≤ 0.05

*BMI* Body mass index, *WC* Waist circumference, *WHR* Waist, hip ratio,, *FBS* Fasting blood sugar, *TG* Triglyceride, *T-chol* Total cholesterol, *HDL-C* High density lipoprotein cholesterol, *LDL* Low-density lipoprotein, *SBP* Systolic blood pressure, *DBP* Diastolic blood pressure, *PAI-1* Plasminogen activator inhibitor type-1, *MCP-1* Monocyte chemoattractant protein-1, *hs-CRP* High-sensitivity C-reactive protein, *TGF-β* Transforming growth factor beta, *RMR* Resting metabolic rate

*P*-value was for t test and *P*-value* was for ANCOVA, adjusted for age, BMI, physical activity, energy, *P ≤ 0/05 is significant

### Association between DPI and RMR.per.kg in participants

The association between DPI and RMR/kg is displayed in the crude and two adjusted binary regression models in Table 4. In crude model, there was a marginally significant association between DPI and RMR.per.kg (OR = 1.41, 95% CI = 0.94–2.11, *P* = 0.09). However, in model 1 after adjusting for age, WHR, energy intake and PA, there was no significant association (OR = 1.27, 95% CI = 0.74–2.18, *P* = 0.37). But in model 2, after adjusting for model 1 plus all components of dietary intakes that were significant after adjustment, marital status, housing status, job, weight loss history in past years, number of family members, economic status, blood pressure and education the results showed that woman with higher adherence to DPI increased odds of RMR.per.kg (≥ 20 kcal/24 h/kg) (OR = 2.77, 95% CI = 0.98—7.82, *P* = 0.05), on the other words had lower odds of hypometabolic status (RMR/kg < 20 kcal/24 h/kg).

**Table 4:** Relation between adherence DPI and RMR. Per. kg

		RMR.per.kg		
		OR (95% CI)	β ± SE	*P*-value*
Crude model	Low adherence of DPI	1^*^	1	0.09
	High adherence of DPI	1.41(0.94–2.11)	0.34 ± 0.20	
*Adjusted*				
Model 1	Low adherence of DPI	1	1	0.37
	High adherence of DPI	1.27(0.74–2.18)	0.24 ± 0.27	
Model 2	Low adherence of DPI	1	1	**0.05**
	High adherence of DPI	2.77(0.98–7.82)	1.02 ± 0.52	

Bold: *P* value ≤ 0.05

*OR* Odds ratio; *CI* Confidence interval, *SE* Standard error, *DPI* Dietary phytochemical index, *RMR* Resting metabolic rate

Binary logistic regression; Crude model and adjusted models, Crude model; Unadjusted, model 1; Adjusted for age, WHR, energy intake, and physical activity, model 2; adjusted for all variables model 1 Plus all components of dietary intakes that were significant after adjustment, marital status, housing status, job, weight loss history in past years, number of family members, economic status, blood pressure and education

^*^P ≤ 0/05 is significant, ^*^Considered as reference; RMR.per.kg < 20 kcal/24 h/kg considered as reference group

### Mediatory effect of inflammatory markers on association between RMR.per.kg and DPI

We assessed the effect of inflammatory markers including; PAI-1, TGF-β, MCP-1 and hs-CRP as mediatory markers for the significant association between DPI and RMR.per.kg. (Table 5). By including each of these inflammatory markers as a confounding variable along with the other confounders in the final model, which showed a significant association between DPI and RMR.per.kg. We observed that inflammatory markers including: PAI-1 (OR = 0.98, 95% CI − 0.10–7.98, *P* = 0.95), TGF-β (OR = 0.83, 95% CI 0.09–7.67, *P* = 0.87) and MCP-1 (OR = 3.11, 95% CI 0.92–10.50, *P* = 0.06) eliminated this significance so they can be considered as mediatory markers. Although, the results shown that hs-CRP cannot be considered as mediatory markers because remain this significance (OR = 3.11, 95% CI 0.92–10.50, *P* = 0.04).

**Table 5:** The association of the mediating effect of some inflammatory markers

		RMR.per.kg		
		OR (95% CI)	β ± SE	*P*-value*
PAI-1(mg/dI)	Low adherence of DPI	1^*^	1	**0.95**
	High adherence of DPI	0.98 (− 0.10–7.98)	− 0.06 ± 1.09	
MCP-1(mg/dI)	Low adherence of DPI	1	1	**0.06**
	High adherence of DPI	3.11 (0.92–10.50)	1.13 ± 0.62	
hs-CRP (mg/L	Low adherence of DPI	1	1	0.04
	High adherence of DPI	4.0 (1.02–15.62)	1.38 ± 0.69	
TGF-β(mg/L)	Low adherence of DPI	1	1	**0.87**
	High adherence of DPI	0.83 (0.09–7.67)	− 0.18 ± 1.13	

Bold: *P* value ≤ 0.05

*OR* Odds ratio, *CI* Confidence interval, *SE* Standard error, *DPI* Dietary phytochemical index, *PAI-1* Plasminogen Activator Inhibitor type-1, *MCP-1* Monocyte chemoattractant protein-1, *TGF-β* Transforming growth factor beta, *hs-CRP* High sensitivity c-reactive protein, *RMR* Resting metabolic rate

Binary logistic regression adjusted, the inflammatory markers as covariate in final model. Adjusted for age,WHR, energy intake, and physical activity, all components of dietary intakes that were significant after adjustment, marital status, housing status, job, weight loss history in past years, number of family members, economic status, blood pressure and education as covariates in addition to the inflammatory markers

^*^Considered as reference; RMR.per.kg < 20 kcal/24 h/kg group considered as reference

## Discussion

In this study, we assessed any potential association between DPI and RMR, mediated by inflammatory factors (MCP-1, PAI-1, TGF-β and hs-CRP), in overweight and obese women. The findings of this study provide novel insight into the relationship between adherence to DPI and reducing the chance of developing hypo-metabolism and mediation of inflammatory factors (MCP-1, PAI-1 and TGF-β) in obese and overweight women. By modulating the effect of other confounders, this association remained, and no mediating effect was observed for hs-CRP. Previous studies have shown that the quality of a diet is positively associated with obesity and overweight, which is consistent with the present study [41]. Indeed, our study indicated that there was a positive association between RMR and high phytochemical diets in adult women. DPI, which includes fruits, vegetables, grains, legumes, seeds, nuts, soybeans and soybean products, can impact on RMR due to their fiber, vitamin, and mineral content [26]. In agreement with our study, Jones et al. reported that diets rich in oleic acid from olive oil may increase energy intake. Polyphenols, such as coffee (caffeine), teas (caffeine, catechins) spices (curcumin, capsaicinoids, piperine), raspberry (ketones), berries and cherries (anthocyanin), grapes (resveratrol) chocolate (cocoa), citrus (alkaloid synephrine), due to the increase of thermogenesis and secretion of catecholamines from the adrenal medulla, as well as post-ganglion fibers of the sympathetic nervous system which stimulate adrenergic receptors in the liver and adipose tissue, can increase the resting metabolic rate, and thus affect weight loss [19, 23]. Jayarathne et al. indicated the protective effects of anthocyanin in obesity-associated inflammation and changes in gut microbiome [42]. Indeed, polyphenols in fruits, vegetables, legumes, nuts, and seeds that have antioxidant properties have an inhibitory effect on nuclear transcription factor KB (NF-KB), the major transcription factor in inflammation, inhibiting tumor necrosis factor-alpha (TNF-α) expression, which stimulates the expression of cyclooxygenase (COX) -2, and also by increasing adiponectin and peroxisome-proliferator activated receptor gamma (PPARɣ), leading to a reduction in inflammatory markers [43, 44]. In this study, adherence to DPI was significantly associated with a reduction in inflammatory markers (MCP-1، PAI-1 and TGF-β) and an increase the RMR through the mediation of inflammatory factors. Indeed, DPI, mainly due to the high content of unsaturated fatty acids, vitamins, and minerals, with antioxidant, anti-inflammatory, and appetite reducing properties, plant protein, fiber, and regulation of carbohydrates, lipids and fat cell metabolism, induce their beneficial effects [45, 46]. Several studies have shown that vitamins, minerals, and fiber are effective in reducing hypometabolism due to their antioxidant and anti-inflammatory properties, and, as a result, they can facilitate weight loss. Our results showed that PAI-1 and TGF-β had a significant mediatory effect, but MCP-1 was only marginally significant, between RMR and DPI. However, other inflammatory factors, such as hs-CRP, did not affect the association between RMR and DPI. A prior study has shown the association between DPI and RMR may be mediated by inflammatory markers (MCP-1, PAI-1 and TGF-β) [39]. Several possible explanatory mechanisms can be considered here. The first is that polyphenols in the DPI reduce TNF-α, where TNF-α can directly affect fat cells to regulate leptin secretion and stimulates the secretion of inflammatory markers (MCP-1, PAI-1 and TGF-β) [47–49]. On the other hand, reducing TNF-α reduces the secretion of inflammatory markers and leptin, which decreases leptin increases the expression of uncoupling proteins (UCPs) (UCP-1,UCP-2 and UCP-3) in adipose tissue and skeletal muscle, UCPs are mitochondrial inner membrane proteins that play an important role in the energy expenditure of the whole body [35, 50]. Leptin may directly increase glucose uptake and fatty acid oxidation in skeletal muscle and adipose tissue [36]. The second plausible mechanism is that vitamins and minerals in the DPI that have antioxidant properties that can reduce TNF-α, which consequently redcues inflammatory markers as well as ROS in mitochondria [28, 29]. moreover, the vitamins and minerals in DPI can lead to an increase in adenosine triphosphate (ATP) production, which can eventually elicit an increase in RMR [29]].

The strengths of this study are the adjustment of all known confounders, such as age and total energy intake, physical activity, and waist circumference, the high sample size, and, to our knowledge, it is the first study to evaluate the relationship between DPI and RMR mediated by inflammatory factors in overweight and obese women. The limitations of the present study include the evaluation method of measuring DPI, where, in this method, the amount of phytochemicals received was determined based on the intake of foods rich in phytochemicals and energy intake, and we were unable to measure the actual amount of phytochemicals received. Moreover, due to the cross-sectional nature of the study, causal inferences cannot be drawn. Finally, although we used a valid and reliable FFQ for assessing dietary intakes, the potential for recall and misclassification bias cannot be entirely ameliorated.

## Conclusion

The results of the present study showed that adherence to a high phytochemical diet may decrease the chance of developing hypometabolism, by reducing inflammatory factors (MCP-1, PAI-1 and TGF-β) that have a mediating effect. Nevertheless, due to the paucity of supporting evidence, the authors advocate that further prospective and interventional studies on a larger scale would be advantageous in further explaining this relationship.

## Data Availability

The datasets used and/or analysed during the current study available from the corresponding author on reasonable request.

## Abbreviations

DPI: Dietary phytochemical index
RMR: Resting metabolic rate
FFQ: Food frequency questionnaire
TGF-β: Transforming growth factor-beta
MCP-1: Monocyte chemoattractant protein-1
PAI-1: Plasminogen activator inhibitor-1
CVD: Cardiovascular disease
WHO: World health organization
TG: Triglyceride
hs-CRP: High-sensitivity C-reactive protein
FFM: Fat-free mass
USDA: United States Department of Agriculture
PA: Physical activity
IPAQ: International physical activity questionnaire-short form
METs-h / week: Metabolic equivalent hours per week
WHR: Waist to hip ratio
COX: Cyclooxygenase
PPARɣ: Peroxisome-proliferator activated receptor gamma
UCPs: Uncoupling proteins
ATP: Adenosine triphosphate
